# Indel detection from Whole Genome Sequencing data and association with lipid metabolism in pigs

**DOI:** 10.1371/journal.pone.0218862

**Published:** 2019-06-27

**Authors:** Daniel Crespo-Piazuelo, Lourdes Criado-Mesas, Manuel Revilla, Anna Castelló, Ana I. Fernández, Josep M. Folch, Maria Ballester

**Affiliations:** 1 Plant and Animal Genomics, Centre for Research in Agricultural Genomics (CRAG), CSIC-IRTA-UAB-UB Consortium, Bellaterra, Spain; 2 Departament de Ciència Animal i dels Aliments, Facultat de Veterinària, Universitat Autònoma de Barcelona (UAB), Bellaterra, Spain; 3 Departamento de Mejora Genética Animal, Instituto Nacional de Investigación y Tecnología Agraria y Alimentaria (INIA), Madrid, Spain; 4 Departament de Genètica i Millora Animal, Institut de Recerca i Tecnologia Agroalimentàries (IRTA), Caldes de Montbui, Spain; Huazhong Agriculture University, CHINA

## Abstract

The selection in commercial swine breeds for meat-production efficiency has been increasing among the past decades, reducing the intramuscular fat content, which has changed the sensorial and technological properties of pork. Through processes of natural adaptation and selective breeding, the accumulation of mutations has driven the genetic divergence between pig breeds. The most common and well-studied mutations are single-nucleotide polymorphisms (SNPs). However, insertions and deletions (indels) usually represents a fifth part of the detected mutations and should also be considered for animal breeding. In the present study, three different programs (*Dindel*, *SAMtools mpileup*, and *GATK*) were used to detect indels from Whole Genome Sequencing data of Iberian boars and Landrace sows. A total of 1,928,746 indels were found in common with the three programs. The *VEP* tool predicted that 1,289 indels may have a high impact on protein sequence and function. Ten indels inside genes related with lipid metabolism were genotyped in pigs from three different backcrosses with Iberian origin, obtaining different allelic frequencies on each backcross. Genome-Wide Association Studies performed in the *Longissimus dorsi* muscle found an association between an indel located in the C1q and TNF related 12 (*C1QTNF12*) gene and the amount of eicosadienoic acid (C20:2(n-6)).

## Introduction

Pork is one of the world’s most produced meat. Selective breeding in pigs has been developed in parallel to the increase and intensification of this productive sector. Over the last decades, genetic selection has notably improved meat-production efficiency in commercial pig breeds. However, this artificial selection had the unwanted drawback of reducing the pork sensorial and technological properties of meat. These modifications were driven by the reduction of intramuscular fat (IMF) content and fatty acid (FA) composition changes [1].

Commercial breeds as Landrace possess an efficient meat production with a rapid growth and a leaner carcass, but the resulting meat has lower IMF and higher polyunsaturated FAs (PUFA) content compared with some indigenous pig breeds, such as the Iberian pig [2]. The Iberian breed is characterized by its higher IMF content with a great proportion of monounsaturated FAs (MUFA) [3]. In addition, MUFA have a more oxidative stability than PUFA, improving the organoleptic properties of meat [4]. In contrast, PUFA consumption, in particular omega-3, has the beneficial role of decreasing the total cholesterol concentration, while saturated FAs (SFA) increase the risk of suffering cardiovascular diseases [5,6].

Fatty acid composition in muscle is determined by physiological conditions such as fed and fasted states [7], environmental factors such as nutrition [4,8] and genetic factors; carcass and FA composition traits in pigs that range from moderate to high heritability values [9–12].

The genetic divergence between breeds has been driven by the accumulation of mutations through processes of natural adaptation to the environment and selective breeding. Genetic mutations can be produced by base pair substitution, but also by insertion, inversion, fusion, duplication or deletion of DNA sequences. The development of next generation sequencing (NGS) technologies has improved the detection of these genomic variants. Hitherto, the most well-known variants studied with this method have been the substitutions of single nucleotide polymorphisms (SNPs), which represent almost the 80% over all the detected variants [13–15]. In contrast, insertions and deletions (indels) have been less characterized although the genome-wide ratio of indels to SNPs has been estimated as 1 indel for every 5.3 SNPs [16]. Studies in *Drosophila melanogaster* and *Caenorhabditis elegans* have determined that indels represent between 16% and 25% of all genetic polymorphisms in these species [17,18]. In addition, studies performed in humans and chimpanzees evidenced that indels instead of SNPs were the major source of evolutionary change [19–21].

As it has been described over the last decades, the most frequently found indel was the 1 base pair (bp) long [22,23]. Furthermore, a major proportion of deletions than insertions was observed in the genome of 18 mammals, with the exception of the opossum [24]. A mechanism that favours the occurrence of deletions was proposed by de Jong & Rydén [25], in which the loops formed by slipped mispairing after DNA strand breakage are trimmed off. In pigs, recent studies using whole genome sequencing (WGS) have detected the 1 bp long indel as the most frequent indel, but the deletion/insertion ratios differ [26–29].

Indels can produce frameshifts in the reading frame of a gene or modify the total number of amino acids in a protein, but they can also affect gene expression levels. In pigs, indels were found to affect backfat thickness [30] and fat deposition [31] through the alteration of gene expression, underlining the importance of these variants for animal production.

The objectives of this study were to identify indels from WGS data of Iberian and Landrace pigs, which were founders of an experimental cross (IBMAP) with productive records for FA composition, and to study the association between a selection of indels and meat quality traits in three different genetic backgrounds.

## Material and methods

### Ethics statement

The present study was performed in accordance with the regulations of the Spanish Policy for Animal Protection RD1201/05, which meets the European Union Directive 86/609 about the protection of animals used in experimentation. All experimental procedures followed national and institutional guidelines for the Good Experimental Practices and were approved by the IRTA (*Institut de Recerca i Tecnologia Agroalimentàries*) Ethics Committee.

### Animal material and phenotypic records

The pigs used in this study belonged to the Iberian and Landrace breeds. The Iberian line, called Guadyerbas, is a unique black hairless line that has been genetically isolated in Spain since 1945 [32]. The Landrace line belonged to the experimental farm Nova Genètica S.A. (Lleida, Spain). WGS data of seven founders of the IBMAP experimental population [32], two Iberian boars and five Landrace sows, were used for indel detection. Analysis of indel segregation and association with meat quality traits were performed in 441 individuals of different backcrosses: 160 BC1_LD ((Iberian x Landrace) x Landrace), 143 BC1_DU ((Iberian x Duroc) x Duroc) and 138 BC1_PI ((Iberian x Pietrain) x Pietrain). All animals were reared in the experimental farm of Nova Genètica S.A. (Lleida, Spain). Population structure of these three backcrosses is depicted in S1 Fig.

Animals were fed *ad libitum* with a cereal-based commercial diet and slaughtered at an average age of 179.8 ± 2.6 days with an average carcass weight of 72.2 kg. Blood from founder animals was collected in 4 ml EDTA vacutainer tubes and stored at -20°C until analysis. Samples of diaphragm tissue were collected from backcrossed animals, snap-frozen in liquid nitrogen and stored at -80°C until analysis. Genomic DNA was extracted from all samples by the phenol-chloroform method [33].

At the slaughterhouse, 200 g of *Longissimus dorsi* muscle samples were collected from the three backcrosses. The IMF composition was measured with a protocol based on gas chromatography of methyl esters as described in Pérez-Enciso *et al*. [32]. In total, 20 traits were analysed: 17 intramuscular FAs and 3 FA metabolism indices (Table 1). Data values were normalized applying a log_2_ transformation when needed.

**Table 1:** Descriptive statistics including mean and SD of fatty acid composition and FA indices in the *Longissimus dorsi* muscle of the merged dataset and the three backcrosses.

Group	Trait	Name	Merged dataset		BC1_DU		BC1_LD		BC1_PI	
			Mean	SD	Mean	SD	Mean	SD	Mean	SD
**SFA**	C14:0	Myristic acid	1.17	0.24	1.27	0.23	1.18	0.15	1.06	0.27
	C16:0	Palmitic acid	22.99	1.51	23.94	1.65	22.59	1.20	22.42	1.14
	C17:0	Margaric acid	0.27	0.10	0.25	0.10	0.27	0.07	0.30	0.13
	C18:0	Stearic acid	14.21	1.44	14.33	1.75	14.18	1.03	14.13	1.46
	C20:0	Arachidic acid	0.26	0.11	0.23	0.08	0.26	0.12	0.31	0.12
										
**MUFA**	C16:1(n-9)	7-Hexadecenoic acid	0.36	0.11	0.31	0.12	0.39	0.09	0.39	0.10
	C16:1(n-7)	Palmitoleic acid	2.61	0.50	2.81	0.52	2.50	0.39	2.53	0.52
	C17:1	Heptadecenoic acid	0.23	0.10	0.19	0.09	0.27	0.11	0.23	0.10
	C18:1(n-9)	Oleic acid	37.08	5.78	35.97	5.63	40.07	2.77	35.15	6.92
	C18:1(n-7)	Vaccenic acid	3.91	0.33	3.83	0.30	3.88	0.36	4.03	0.30
	C20:1(n-9)	Gondoic acid	0.82	0.20	0.73	0.16	0.85	0.11	0.88	0.26
										
**PUFA**	C18:2(n-6)	Linoleic acid	11.92	5.01	12.11	5.83	10.36	2.38	13.32	5.64
	C18:3(n-3)	α-Linolenic acid	0.50	0.23	0.40	0.13	0.65	0.29	0.44	0.14
	C20:2(n-6)	Eicosadienoic acid	0.51	0.14	0.43	0.12	0.54	0.12	0.57	0.14
	C20:3(n-3)	Eicosatrienoic acid	0.22	0.13	0.18	0.10	0.20	0.15	0.28	0.13
	C20:3(n-6)	Dihomo-γ-linolenic acid	0.43	0.28	0.45	0.29	0.28	0.13	0.58	0.29
	C20:4(n-6)	Arachidonic acid	2.53	2.04	2.57	1.94	1.54	0.74	3.49	2.53
										
**Metabolic Ratios**	SFA	Saturated fatty acids	38.90	2.44	40.01	3.13	38.47	1.64	38.17	1.84
	MUFA	Monounsaturated fatty acids	44.78	6.25	43.54	6.05	47.95	3.07	42.81	7.54
	PUFA	Polyunsaturated fatty acids	15.88	7.26	15.96	8.12	13.37	3.30	18.37	8.37

### Whole genome sequencing

The whole genome of seven founders of the IBMAP population was sequenced at CNAG (National Centre for Genome Analysis, Barcelona, Spain) on an Illumina HiSeq2000 instrument (Illumina, San Diego, CA, USA). Paired-end sequencing libraries, with approximately 300 bp insert size, were generated using TruSeq DNA Sample Prep Kit (Illumina, San Diego, CA, USA). For each sample, around 40 million 100 bp-long paired-end reads were produced with an average sequencing depth of 11.7x. Whole genome sequencing files of the seven BC1_LD founders are described in Revilla *et al*. [34] and were deposited in the NCBI Sequence Read Archive (SRA) under accession nos. SRR5229970, SRR5229971, SRR5229972, SRR5229973, SRR5229974, SRR5229975 and SRR5229976.

Sequences were trimmed based on their quality using the FastQC [35] software. Then, reads were mapped against the reference genome sequence assembly (*Sscrofa10*.*2*) using the Burrows-Wheeler Alignment (BWA) tool [36]. Duplicated reads or those which were under a Phred-based quality score of 20 were removed. Finally, alignment result files (in bam format) were prepared for indel detection.

### Indel detection and effects prediction

Several programs allow performing indel calling from WGS bam files. Following the article of Neuman *et al*. [37] on the comparison of short indel detection programs, we applied the recommended pipelines on the use of these three programs: *Dindel* (version 1.01) [38], *SAMtools mpileup* (version 0.1.19) [39], and *Genome Analysis Toolkit* (*GATK*) (version 3.4–46) [40].

The *Variant Effect Predictor* (*VEP*) (version 82) [41] tool of Ensembl (http://www.ensembl.org/) was used to quickly and accurately predict the effects and consequences of indels previously found on Ensembl-annotated transcripts [41]. Furthermore, to predict the possible effect of an indel in the secondary structure of a protein, *JPred4* [42] was used.

Finally, ten indels were selected for indel validation and association analysis if they followed any of these two criteria:

1. those start or stop variants related with lipid metabolism
2. those indels with high or moderate severity that were found at extreme frequencies in the founder animals (IB = 1 & LD≤0.2 or IB = 0 & LD≥0.8). Among this subset of 127 indels, those involved in lipid metabolism were prioritized.

### Genotyping

For indel validation and association analysis, ten indels were genotyped in three experimental backcrosses: BC1_DU (n = 143), BC1_LD (n = 160) and BC1_PI (n = 138), using Taqman OpenArray genotyping plates custom designed in a QuantStudio 12K flex Real-Time PCR System (ThermoFisher Scientific, Waltham, MA, USA).

The same animals of BC1_LD and BC1_PI were genotyped with the Porcine SNP60K BeadChip (Illumina, San Diego, CA, USA), while BC1_DU samples genotypes were obtained with the Axiom Porcine Genotyping Array (Axiom_PigHDv1; Affymetrix, Santa Clara, CA, USA). Only those variants shared by both genotyping platforms were kept. A total of 38,424 SNPs remained after removing SNPs with a minor allele frequency (MAF) < 5% and SNPs with missing genotype > 5% data using *PLINK* (1.90b5 version) [43].

### Genome-Wide association analysis

Genome-Wide Association Studies (GWAS) were performed between the measured phenotypes of IMF composition and the previously genotyped variants of the three backcrosses (38,424 SNPs and nine indels) along the pig reference genome assembly (*Sscrofa11*.*1*). The studies were conducted with *GEMMA* [44] following the mixed linear model:

$$y_{ijklm}=Sex_{i}+Batch_{j}+Backcross_{k}+\beta c_{l}+u_{l}+\delta_{l}a_{m}+e_{ijklm},$$

where y_ijklm_ indicates the value of the phenotypic observation in the l^th^ individual; sex (two categories), batch (fourteen categories) and backcross (three categories) are fixed effects; β is a covariate coefficient with c being carcass weight; u_l_ is the infinitesimal genetic random effect and distributed as N(0, Kσ_u_), where K is the numerator of the kinship matrix; δ_l_ represents the allelic effect, calculated as a regression coefficient on the l^th^ individual genotype for the m^th^ SNP or indel (values -1, 0, +1); a_m_ represents the additive effect associated with the m^th^ SNP or indel; and e_ijklm_ is the random residual term. Genomic kinship was obtained selecting the “-gk 1” option in *GEMMA* software [44], which calculates a centred relatedness matrix using the genotypic information of the individuals.

GWAS were also performed individually for each one of the three backcrosses following the previously described model, except for the fixed effect of the backcross which was removed from the model.

The multiple test correction was conducted with the *p*.*adjust* function incorporated in R (www.r-project.org) using the false discovery rate (FDR) method developed by Benjamini and Hochberg [45]. In order to consider a SNP or an indel as significant or suggestive a cut-off was set at FDR≤0.05 or FDR≤0.1, respectively.

## Results and discussion

### Genome-wide detection of indels in Iberian and Landrace animals

**Fig 1:**
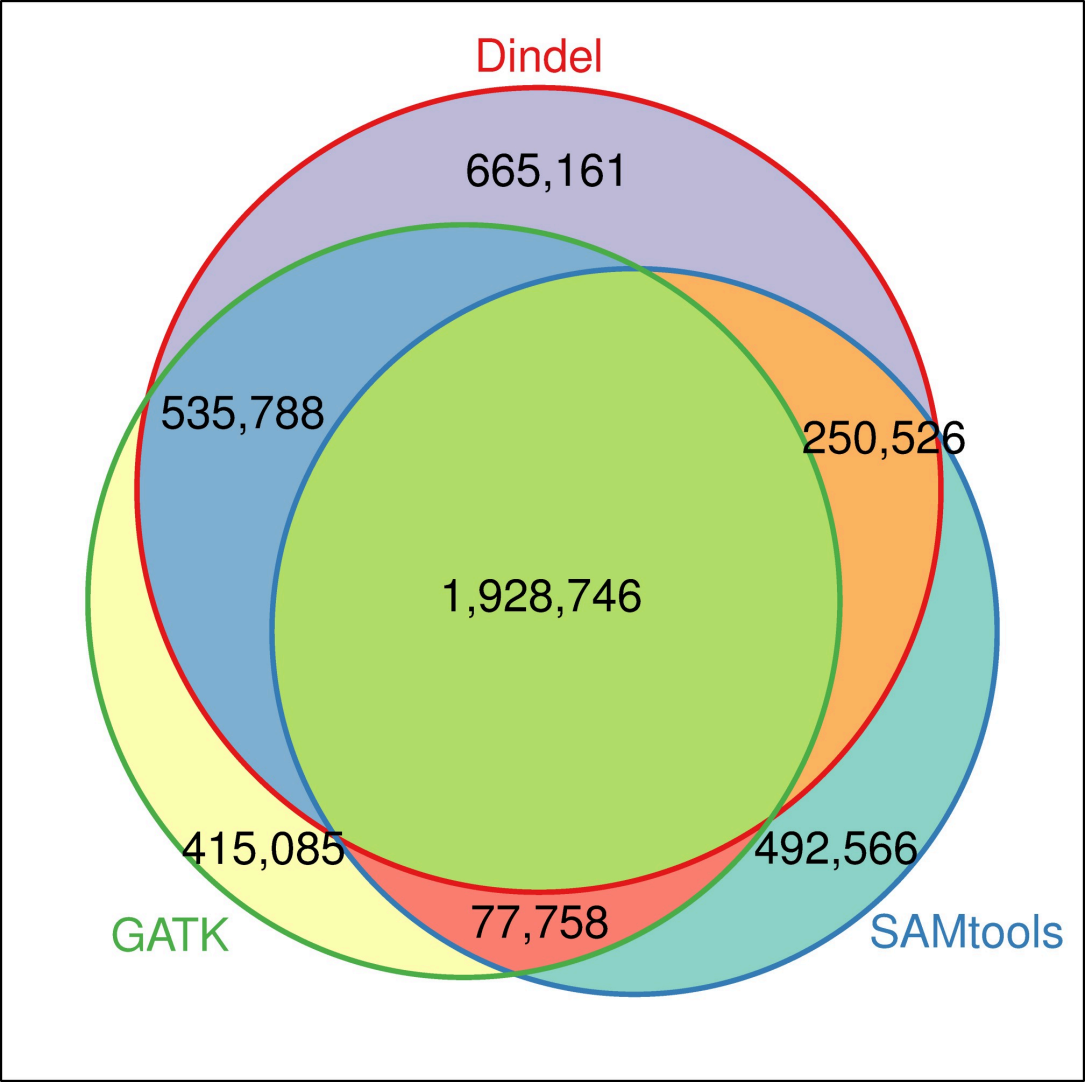
Weighted Venn diagram showing the number of indels shared between the three indel detection programs: *Dindel*, *Pindel* and *SAMtools mpileup*. A total of 1,928,746 indels were found in common.

Whole genome sequencing data of seven founders of the IBMAP population (two Iberian boars and five Landrace sows) were used for indel detection with *Dindel*, *SAMtools mpileup* and *GATK* software. *Dindel* was the program that detected the highest number of indels (3,380,221) as opposed to *SAMtools mpileup* and *GATK* (2,749,596 and 2,957,377, respectively). To reduce the rate of false positives, only indels (1,928,746) that were found in common between the three programs were considered for further analyses (Fig 1). In addition, 50,528 indels were discarded for not displaying the same genotype in at least two programs.

Repetitive elements, such as microsatellites, are short insertions or deletions that can interfere with the detection and annotation of indels. Thus, to reduce the interference of repetitive elements in the next steps, 105,783 variants were discarded if they were triallelic or the alternative allele was different among individuals for the same chromosomal position. Moreover, 141,391 indels were trimmed because they were homozygous for the alternative allele in all samples and may not be segregating in our population. Hence, we only considered the final list comprising 1,631,044 indels for further analysis (S1 Table).

In a preliminary study of our group, in which SNP calling was performed from WGS of these seven IBMAP founders, the number of SNPs identified after the quality filter was 4.9 million in the Iberian boars and 6 million in the Landrace sows. Therefore, the number of indels detected (1.6 million indels) was within the expected range (16–25%) of the total number of variants detected [13–18]. Nevertheless, another study in pigs reported that indels were less frequent than SNPs in a proportion of 1 to 10 [26].

**Fig 2:**
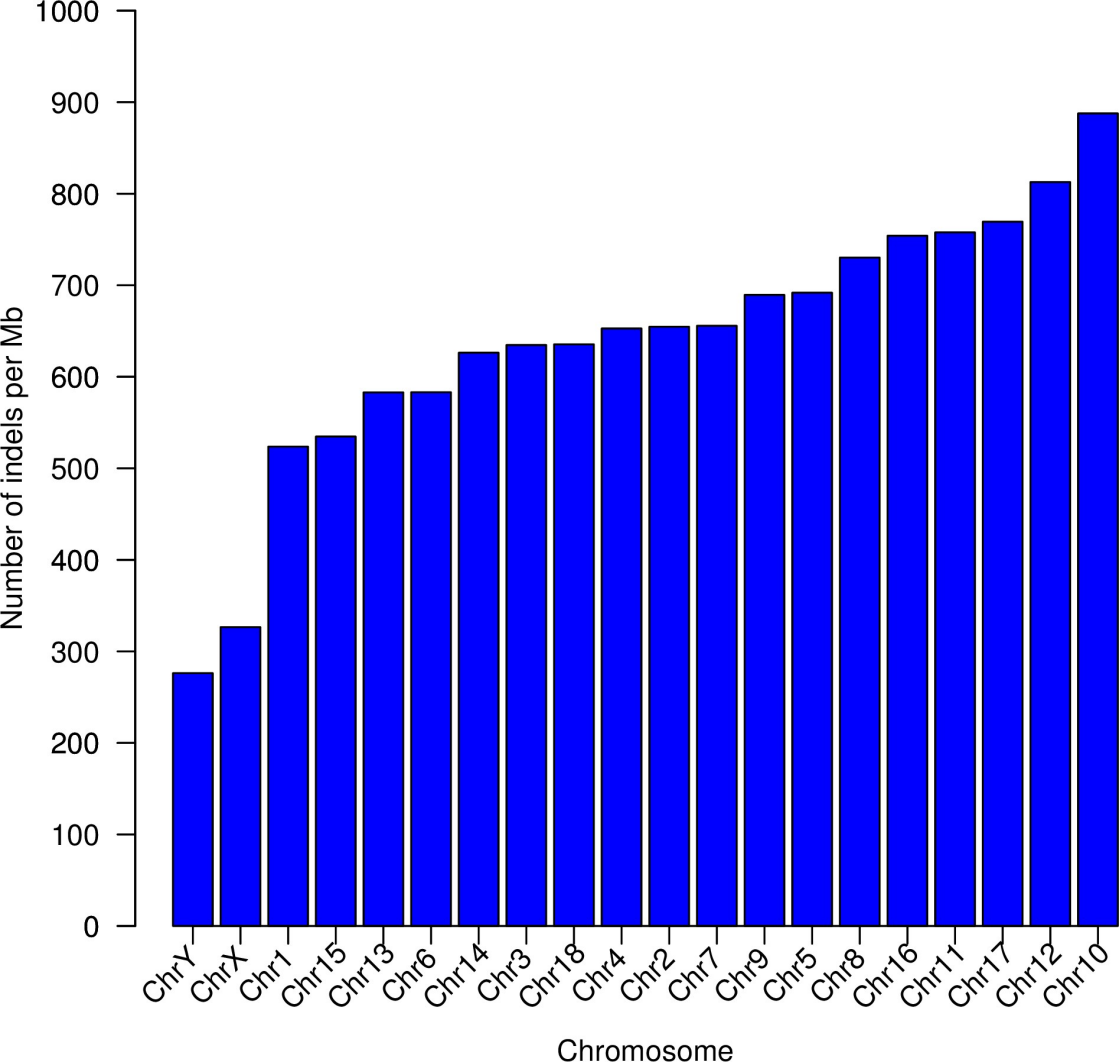
Distribution of the density of indels across chromosomes calculated as number of indels per Mb. Chromosomes are sorted in increasing order of density value.

The distribution of the indels found along all the *Sus scrofa* chromosomes (SSC) showed that sexual chromosomes (SSCX and SSCY) had lower density of indels than autosomes (Fig 2). Disregarding the pseudoautosomic regions, this low density of indels in the sexual chromosomes is probably caused by the low recombination rate, only possible for the X chromosome in females, and by the appearance of hemizygous recessive lethal mutations in males. In addition, males present one copy of each heterosome, and accordingly, the density of mutations in autosomes, which have two copies of each chromosome, is higher than in heterosomes. The autosome that had the highest density of indels was SSC10, while SSC1 had the lowest (Fig 2).

**Fig 3:**
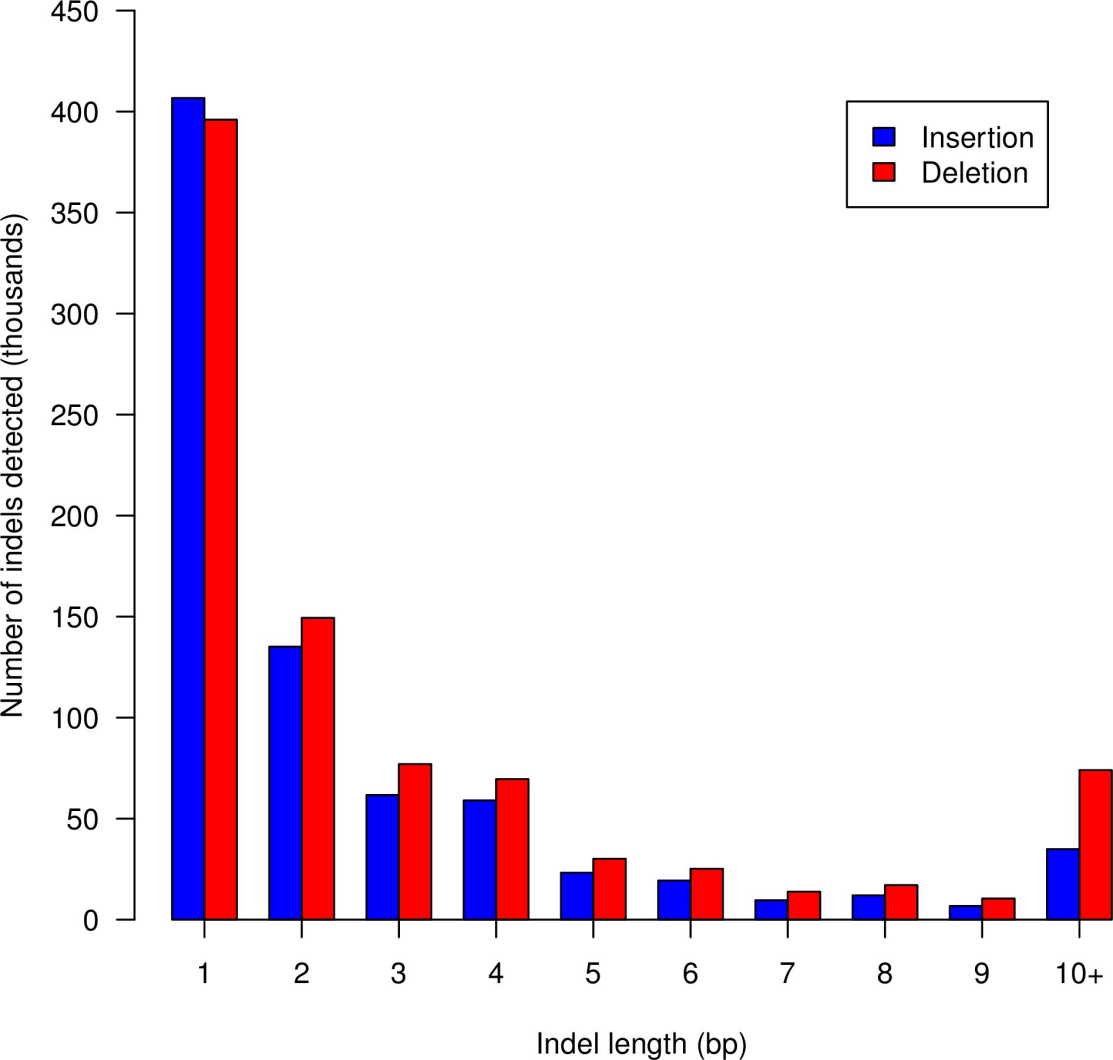
From the total of 1,631,044 indels detected, it is represented the quantity of them according to their length in bp. Insertions are in red and deletions are in blue.

In accordance with the literature, indel frequencies decreased as their length increased [27,46] and thus, 1 bp long indel was the most frequent indel found (Fig 3), either insertion or deletion [22,23]. Insertions were more frequent than deletions in single bp indels, but from the 1.6 million indels, 52.9% were deletions from 1 to 54 bp and the rest were insertions (47.1%) from 1 to 32 bp. Therefore, deletions were found to be more frequent than insertions, which has been previously reported by some other studies made in pigs [26,28] and follows the mutational mechanisms described by de Jong & Rydén (1981).

### Consequence and severity predictions of the indels detected

The effects (consequence type and severity) of the 1.6 million indels were estimated by the *VEP* platform and are summarized in Table 2. Since a variant may co-locate with more than one transcript, one line of output was provided for each instance of co-location and thus, there were more lines written (1,790,722) than indels entered (1,631,044). In addition, the total number of predicted effects was 1,809,798 as some indels can result in more than one effect in the same transcript (e.g., an indel could cause a frameshift along with a stop gained). Around the third part of the 1.6 million indels (33.1%) did not fall within intergenic regions (539,920 indels) and only 1,758 indels were inside a coding region (0.11%). Finally, the *VEP* platform classified the 1.6 million indels by their possible severity as high (1,289), moderate (561) or low (1,018) impact, and the rest of indels were considered as modifiers.

**Table 2:** Consequences predicted by the *VEP* platform.

Consequence type	Quantity	*VEP* severity
**Total of indels processed (input)**	**1,631,044**	-
intergenic variant	1,091,124	Modifier
intron variant	506,323	Modifier
downstream gene variant	91,517	Modifier
upstream gene variant	90,313	Modifier
non coding transcript variant	11,535	Modifier
3’ UTR variant	7,618	Modifier
NMD transcript variant	5,911	Modifier
splice region variant	1,443	Low
frameshift variant	1,246	High
5’ UTR variant	1,112	Modifier
non coding transcript exon variant	650	Modifier
inframe deletion	359	Moderate
inframe insertion	285	Moderate
coding sequence variant	115	Modifier
splice acceptor variant	94	High
splice donor variant	74	High
mature miRNA variant	41	Modifier
start lost	14	High
stop gained	9	High
protein altering variant	6	Moderate
stop retained variant	5	Low
stop lost	3	High
incomplete terminal codon variant	1	Low

### Indel selection for genotyping

From the total of indels with high and moderate impact (1,850), ten indels were selected to be genotyped in three different genetic backgrounds. These indels were chosen regarding their possible consequence, if they were inside genes that could be related with lipid metabolism and/or considering their frequencies in the founder animals.

Table 3 summarizes the list of genes with indels selected for genotyping:

1. The aspartate beta-hydroxylase (*ASPH*) gene (ENSSSCG00000025087), located on SSC4, contained a predicted frameshift variant (rs691136075) with a high impact. The expression of this gene was found to be negatively correlated with insulin-stimulated sprouting in mice adipose tissue [47].
2. The calpain 9 (*CAPN9*) gene (ENSSSCG00000010182) is located on SSC14 and contained a predicted inframe deletion (rs704351652). *CAPN9* is a member of the calpain family and some of its members have been associated with body fat content and insulin resistance in human and mice [48,49]. This variant was found at extreme frequencies in the founder animals being the alternative allele (*CAPN9*:*c*.*2013_2015delGAA*) fixed in the Iberian boars.
3. The C-C motif chemokine receptor 7 (*CCR7*) gene (ENSSSCG00000017466) is located on SSC12 and contained a predicted frameshift variant (rs789030032). *CCR7* codifies for a chemokine receptor that plays a crucial role in inducing adipose tissue inflammation, insulin resistance and obesity [50,51]. The allele frequency for this indel (*CCR7*:*c*.*1142dupA*) in the Landrace sows was 0.5 while the two Iberian boars were homozygous for the reference allele.
4. The C-reactive protein (*CRP*) gene (ENSSSCG00000021186), located on SSC4, contained a frameshift variant (*CRP*:*c*.*515delT*). High levels of *CRP* has been related with overweight and obesity in human adults [52]. This variant was found fixed in the Iberian boars for the alternative allele (*CRP*:*c*.*515delT*) and the alleles of the Landrace sows were as the reference.
5. The C1q and TNF related 12 (*C1QTNF12*) gene (ENSSSCG00000003333) is located on SSC6 and contained an inframe deletion (*C1QTNF12*:*c*.*557_559delCCG*). This gene is also known as *CTRP12* and *FAM132A*. C1QTNF12 functions as an adipokine that is involved in glucose metabolism and obesity in mice [53,54]. This deletion was found at extreme frequencies in the founders being the alternative allele (*C1QTNF12*:*c*.*557_559delCCG*) fixed in the Iberian boars.
6. The granzyme A (*GZMA*) gene (ENSSSCG00000016903), located on SSC16, contained an inframe insertion (rs792025734). This gene was differentially expressed in the mesenteric adipose tissue of beef cattle with distinct gain [55]. The insertion (*GZMA*:*c*.*129_131dupGTT*) was found with a frequency of 0.8 in the Landrace sows while the Iberian boars were homozygous for the reference allele.
7. The jumonji domain containing 1C (*JMJD1C*) gene (ENSSSCG00000010226) is located on SSC14 and contained an inframe deletion (*JMJD1C*:*c*.*5964_5966delCAG*). *JMJD1C* was found in a human GWAS as a candidate gene for very low-density lipoprotein particles [56]. This variation was found at extreme frequencies in the founders being the alternative allele (*JMJD1C*:*c*.*5964_5966delCAG*) fixed in the Iberian boars.
8. The lysosomal trafficking regulator (*LYST*) gene (ENSSSCG00000010151), located on SSC14, contained an inframe insertion (rs713515754). This gene has been related with hypertriglyceridemia and anomalous lipid and FA composition in the erythrocyte membranes of Chédiak-Higashi human patients [57]. This variation (*LYST*:*c*.*6287_6289dupCCA*) was found with a frequency of 0.8 in the Landrace sows while the Iberian boars were homozygous for the reference allele.
9. The peroxisomal biogenesis factor 19 (*PEX19*) gene (ENSSSCG00000023091) is located on SSC4 and contained a predicted frameshift variant (rs702520311). *PEX19* is assumed to be under regulation by peroxisome proliferator-activated receptor gamma coactivator-1 alpha (PGC-1α) increasing the mitochondrial FA oxidation in human primary myotubes [58]. In addition, peroxisomes are intimately associated with lipid droplets and they are able to perform FA oxidation and lipid synthesis [59]. The frameshift variant was found to be fixed in the Iberian boars for the alternative allele (*PEX19*:*c*.*98_102dupAAGTC*), whereas in the Landrace sows the alternative allele was present with a frequency of 0.2.
10. The sterile alpha motif domain containing 4B (*SAMD4B*) gene (ENSSSCG00000016927), located on SSC16, contained a predicted frameshift variant that causes a stop gained (rs709630954). This gene was found to produce leanness and myopathy in mice due to the dysregulation of the rapamycin complex 1 (mTORC1) signalling [60].

**Table 3:** Selection of the ten genotyped indels with the alternative allele frequency in the Iberian (Freq. IB) and Landrace (Freq. LD) founders and their consequence predicted by the *VEP* platform.

Gene Name	Ensembl Gene ID	Chr.	Position^a^	Reference Allele	Alternative Allele	Freq.^b^ IB	Freq.^b^ LD	Consequence	Severity
*ASPH*	ENSSSCG00000025087^c^	4	78,502,739 (72,104,018)	T	TAGAC	0	0.1	Frameshift variant Stop retained variant	High
*PEX19*	ENSSSCG00000023091	4	98,087,517 (90,197,086)	G	GCAAGT	1	0.2	Frameshift variant	High
*CRP*	ENSSSCG00000021186	4	98,755,304 (90,783,699)	GT	G	1	0	Frameshift variant	High
*C1QTNF12*	ENSSSCG00000003333	6	57,988,405 (63,549,854)	ACCG	A	1	0	Inframe deletion	Moderate
*CCR7*	ENSSSCG00000017466	12	22,151,183 (21,868,256)	T	TA	0	0.5	Frameshift variant Stop retained variant	High
*LYST*	ENSSSCG00000010151	14	59,597,172 (55,547,902)	G	GACC	0	0.8	Inframe insertion	Moderate
*CAPN9*	ENSSSCG00000010182	14	64,251,137 (59,585,649)	GTTC	G	1	0	Inframe deletion	Moderate
*JMJD1C*	ENSSSCG00000010226	14	71,899,504 (66,662,629)	TCTG	T	1	0	Inframe deletion	Moderate
*GZMA*	ENSSSCG00000016903	16	36,388,745 (34,285,211)	G	GTGT	0	0.8	Inframe insertion	Moderate
*SAMD4B*	ENSSSCG00000016927^c^	16	40,171,247 (37,404,073)	TCA	T	0.25	0.7	Frameshift variant Stop gained	High

^a^Position is referred to the indel’s previous nucleotide in the *Sscrofa10*.*2* assembly. Position in the *Sscrofa11*.*1 assembly* is enclosed in parentheses.

^b^Freq. stands for the frequency of the alternative allele.

^c^Novel pig genes that were orthologous with their human, mouse and cow counterpart genes.

### Segregation analysis of the selected indels

The ten selected indels were genotyped in 143 BC1_DU, 160 BC1_LD and 138 BC1_PI individuals. Table 4 shows the genotype frequencies of indels in each backcross. Allele genotyping of the *CRP*:*c*.*515delT* indel failed and this indel was discarded for posterior analysis.

**Table 4:** Genotype frequencies of the nine indels found in each backcross. For each backcross, 143 BC1_DU, 160 BC1_LD and 138 BC1_PI were genotyped.

	***ASPH***			***PEX19***			***C1QTNF12***		
*Alleles*	-/-	ACAG/-	ACAG/ACAG	-/-	AAGTC/-	AAGTC/AAGTC	CCG/CCG	CCG/-	-/-
*BC1_DU*	0.82	0.17	0.01	0.21	0.48	0.31	0.57	0.43	0
*BC1_LD*	0.65	0.32	0.04	0.03	0.25	0.73	0.53	0.47	0
*BC1_PI*	0.92	0.08	0	0	0.03	0.97	0.55	0.45	0
	***CCR7***			***LYST***			***CAPN9***		
*Alleles*	-/-	A/-	A/A	-/-	CCA/-	CCA/CCA	GAA/GAA	GAA/-	-/-
*BC1_DU*	0.60	0.35	0.05	0.73	0.22	0.05	0.43	0.57	0
*BC1_LD*	0.28	0.46	0.26	0.14	0.57	0.29	0.48	0.51	0.01
*BC1_PI*	0.87	0.13	0	0.28	0.55	0.16	0.54	0.46	0.01
	***JMJD1C***			***GZMA***			***SAMD4B***		
*Alleles*	CAG/CAG	CAG/-	-/-	-/-	GTT/-	GTT/GTT	TG/TG	TG/-	-/-
*BC1_DU*	0.54	0.46	0	0.83	0.16	0.01	0.33	0.64	0.04
*BC1_LD*	0.51	0.49	0	0.17	0.45	0.38	0.05	0.50	0.45
*BC1_PI*	0.59	0.41	0	0.30	0.64	0.07	0.13	0.48	0.39

### GWAS results

Nine indels located within genes related with lipid metabolism and genotyped in the three experimental backcrosses were selected for the association analysis. GWAS was performed with a linear-mixed model (*GEMMA* software) among the genotypes of 38,424 SNPs segregating in the three backcrosses and the nine selected indels and the fatty acid composition in muscle.

**Fig 4:**
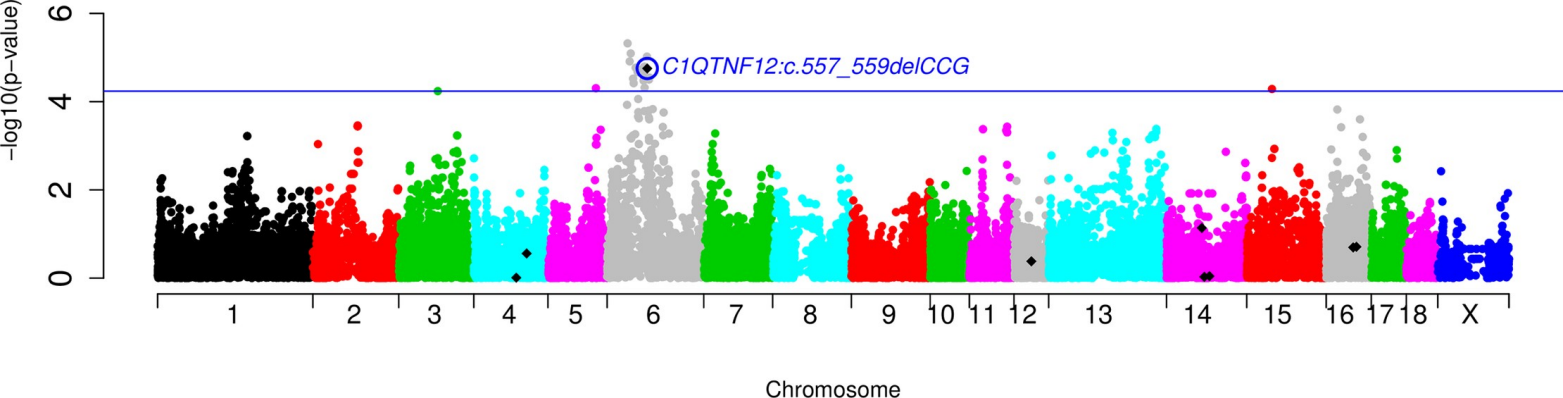
Manhattan plot representing the GWAS analysis for the relative abundance of eicosadienoic acid in the *Longissimus dorsi* muscle of the BC1_PI backcross where the *C1QTNF12* indel (blue circle) was suggestive (FDR≤0.1, blue line). The nine genotyped indels are depicted as black rhombi.

GWAS results in the merged dataset showed no significant association between the nine genotyped indels and the 20 FA composition traits in IMF. However, a suggestive association between the *C1QTNF12*:*c*.*557_559delCCG* indel and the eicosadienoic acid (C20:2(n-6)) (*p*-value = 1.77×10^-5^, FDR = 5.34×10^-2^) was identified in the BC1_PI backcross-specific GWAS (Fig 4). This association was not found in the other two backcrosses BC1_DU (*p*-value = 1.65×10^-1^, FDR = 8.92×10^-1^) and BC1_LD (*p*-value = 1.63×10^-1^, FDR = 9.11×10^-1^) (S2 Fig).

Eicosadienoic acid is the elongated product of linoleic acid, an essential FA that is taken from the diet [61,62] and can be desaturated into arachidonic acid which participates in multiple regulatory pathways [61,62]. The BC1_PI pigs carrying the *C1QTNF12*:*c*.*557_559delCCG* allele had a lower proportion of C20:2(n-6). This result was not observed in the rest of backcrosses despite the *C1QTNF12* indel was segregating at similar frequencies in the three backcrosses (Table 4). We hypothesize that other mechanisms could be modulating the levels of C20:2(n-6) in the BC1_DU and BC1_LD backcrosses and masking the effect of the *C1QTNF12* indel.

*C1QTNF12* is a gene member of the C1QTNF family which preferentially acts in adipose tissue and liver regulating glucose uptake and fatty acid metabolism [54]. C1QTNF12 can also form heterodimers with the protein encoded by the *ERFE* (erythroferrone) gene, another gene member of the C1QTNF family, which is mainly expressed in skeletal muscle and is able to reduce the circulating levels of free FAs without affecting adipose tissue lipolysis [63]. Therefore, alterations of the C1QTNF12/ERFE heterodimer may modify the circulation of free FAs and their accumulation in IMF.

**Fig 5:**
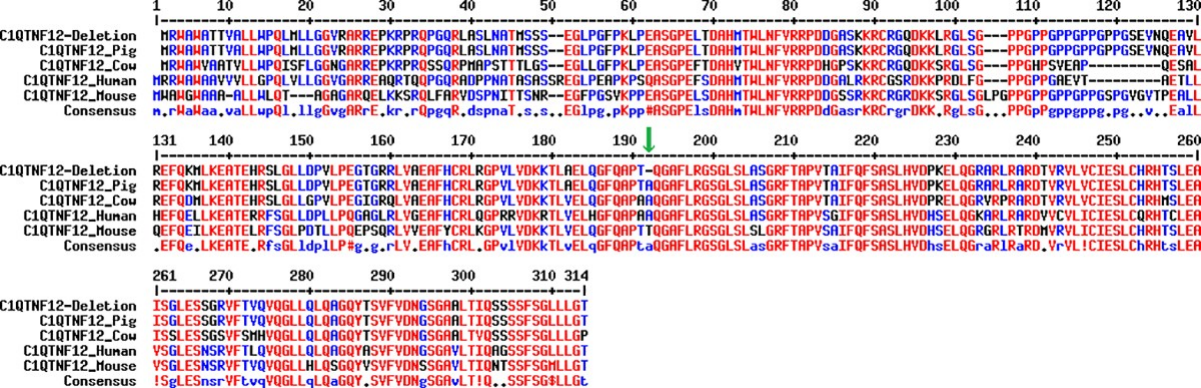
Multiple sequence alignment based on MULTALIN [65] of the porcine C1QTNF12 protein sequence with the deletion and the reference sequences of the C1QTNF12 protein in pig, human, cow and mouse. The green arrow points out the deletion.

**Fig 6:**
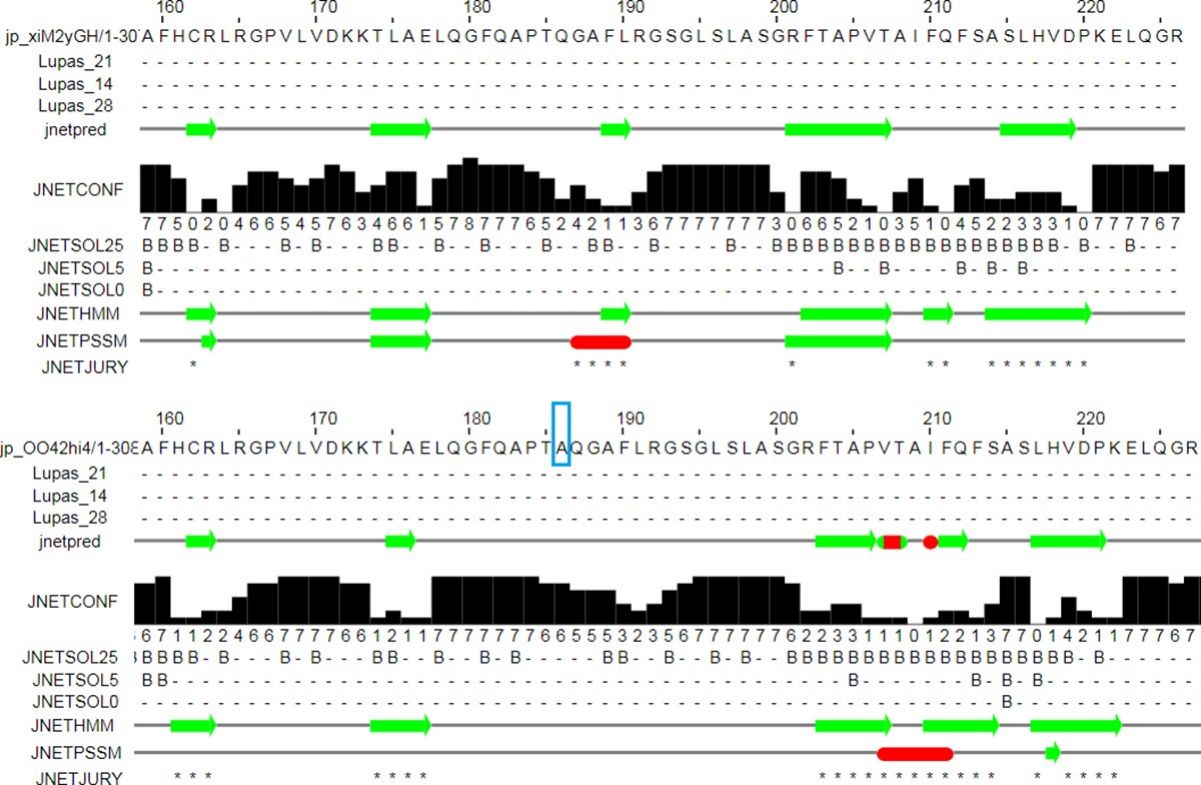
JPred4 prediction of the change in the secondary structure of the porcine C1QTNF12 protein when the alanine in the position 186 (A inside the blue rectangle) of the reference sequence (bottom) is deleted (above). Red segments represent alpha helices and green, beta sheets.

Based on the data from the Ensembl project (www.ensembl.org; release 92) using the *Sscrofa11*.*1* assembly, the porcine *C1QTNF12* gene consists of 8 exons and 7 introns (Ensembl ID: ENSSSCG00000003333). The identified indel produces an inframe deletion of three bases (CCG) in the exon 5 of *C1QTNF12*, which has the consequence of removing the alanine in the position 186 of the final protein. This alanine deletion was located in the C1q/TNF-like domain of C1QTNF12, a domain that is highly conserved among the *C1QTNF12* gene of mammals (Fig 5) and other vertebrate species [64], and is characteristic of the C1QTNF family. Furthermore, the alanine deletion in the position 186 was predicted to cause a new α-helix formation in the secondary structure of C1QTNF12, which could produce an impairment in the protein function (Fig 6).

Nonetheless, the *C1QTNF12* indel was not the most significant genetic variant on SSC6 (Fig 4 and S2 Table). Thus, further studies are required in order to analyse whether other genes or other *C1QTNF12* polymorphisms may be the cause for the differences in the eicosadienoic acid abundance.

In conclusion, in this study we used three different programs that increased the accuracy of indel detection. Nine indels of the 1.6 million indels detected *in silico* were validated through genotyping in three different backcrosses, showing different allelic frequencies. In addition, a suggestive association was found between the *C1QTNF12*:*c*.*557_559delCCG* indel and the eicosadienoic acid abundance. Thus, indels can also be used as genetic markers associated with phenotypic traits of interest.

## Supporting information

S1 Fig Population structure of the three IBMAP experimental backcrosses (BC1_DU, BC1_LD and BC1_PI). (TIF)

S2 Fig QQ-plots and Manhattan plots for the relative abundance of eicosadienoic acid in the *Longissimus dorsi* muscle of the merged dataset and the three backcrosses (BC1_DU, BC1_LD and BC1_PI). The nine genotyped indels are depicted as black rhombi and the *C1QTNF12* indel is encircled in blue. Red and blue lines indicate those polymorphisms that were below the genome-wide significance and suggestive threshold (FDR ≤ 0.05 and FDR ≤ 0.1, respectively).
(TIF)

S1 Table Compressed vcf file containing the 1,631,044 indels found in common between the three programs (*Dindel*, *SAMtools mpileup* and *GATK*) used for predicting the consequence type and severity of the indels by the *VEP platform*. (RAR)

S2 Table GEMMA output for the suggestive (FDR≤0.1) SNPs found in the GWAS analysis for the log2 normalization of the relative abundance of eicosadienoic acid in the *Longissimus dorsi* muscle of the BC1_PI population. (CSV)
