# Supplementary figures and images for: Indel detection from Whole Genome Sequencing data and association with lipid metabolism in pigs
Source: PLoS One. 2019 Jun 27;14(6):e0218862. doi: 10.1371/journal.pone.0218862 (PMC6597088; doi:10.1371/journal.pone.0218862)

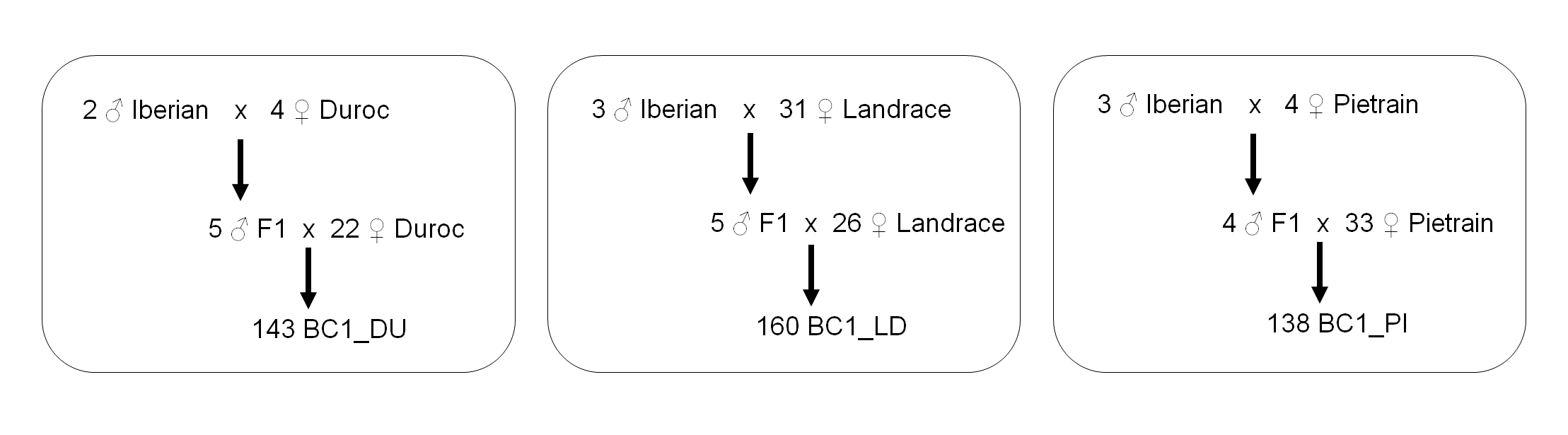

Supplement: S1 Fig — (TIF) [file pone.0218862.s001.tif]

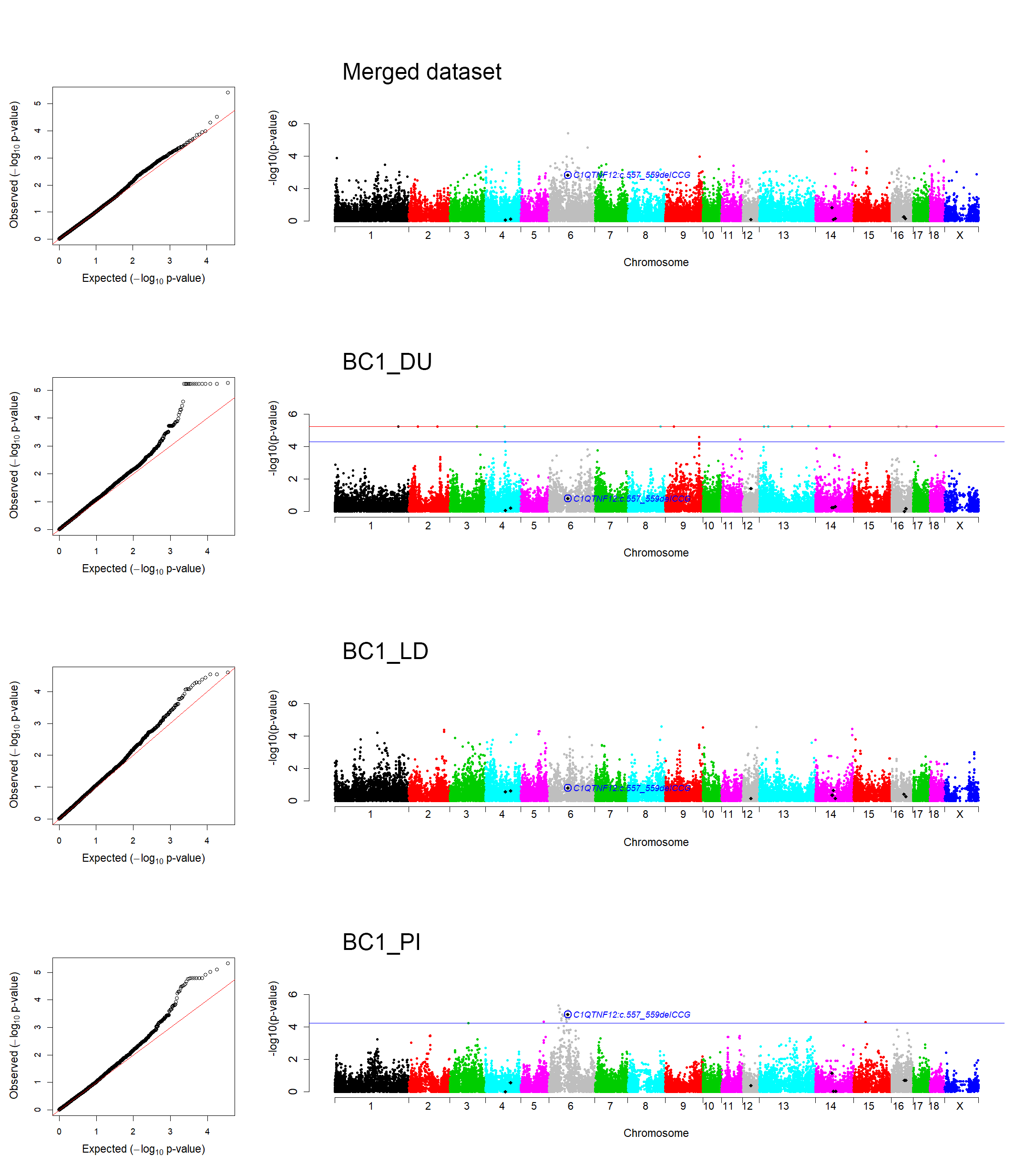

Supplement: S2 Fig — The nine genotyped indels are depicted as black rhombi and the C1QTNF12 indel is encircled in blue. Red and blue lines indicate those polymorphisms that were below the genome-wide significance and suggestive threshold (FDR ≤ 0.05 and FDR ≤ 0.1, respectively). (TIF) [file pone.0218862.s002.tif]
